
JOURNAL INFORMATION
==============================
NLM Title Abbreviation: ScientificWorldJournal
Journal ID: ScientificWorldJournal
NLM Journal ID: 101131163
Journal ID: TSWJ

The Scientific World Journal

ISSN: 2356-6140
EISSN: 1537-744X
Publisher: Wiley

ARTICLE INFORMATION
==============================
PMCID: PMC5917364
PMID: 29861687
DOI: 10.1100/tsw.2006.223
Article ID: 980175
Article version: 1
Subjects: Editorial

In Memory of Joe Wisniewski, Ph.D.: An Outstanding Editor for the Environmental Sciences

Allen Anne Vindenes
ScientificWorld, Ltd., USA
Publication date: 2006
Electronic publication date: 2006 Sep 19
Volume: 6
First page: 1144
Last page: 1145
Copyright: Copyright © 2006 Anne V Allen.
Copyright year: 2006
License: This is an open access article distributed under the Creative Commons Attribution License, which permits unrestricted use, distribution, and reproduction in any medium, provided the original work is properly cited.
License URL: https://creativecommons.org/licenses/by/3.0/

==============================
Editorial
*TheScientificWorldJOURNAL* (2006) 6, 1144-1145
ISSN 1537-744X; DOI 10.1100/tsw.2006.223

TheScientificWorldJOURNAL
www.thescientificworld.com

### In Memory of Joe Wisniewski, Ph.D.: An Outstanding Editor for the Environmental Sciences

Anne Vindenes Allen
*TheScientificWorld, Ltd.*

*Published September 19, 2006*

---

Last month, we heard the shocking news that Joe Wisniewski, the environmental scientist, editor, and entrepreneur, had died. He leaves his wife, Jan, and three young sons.

Joe graduated with a B.S. from the University of Delaware, and then went on to gain an M.S. at Miami and a Ph.D. in environmental sciences at the University of Virginia in 1977. Joe was an Adjunct Professor in the Department of Forestry, North Carolina State University and a world authority on carbon sequestration in forestry. As a researcher, he was a contributor to many journals, including *Science*, *Environmental Management*, *Ambio*, and *Air, Soil and Water Pollution*.

Joe was also a prolific editor for the environmental sciences, editing numerous books and journal special issues from workshops and conferences, and working with publishers, including Kluwer, Springer, and Elsevier. Through his company Wisniewski & Associates, Inc., he also provided management consulting services to industry and government, both in the U.S. and Europe.

I met Joe in 1995 when working as a publishing editor for environmental sciences at Elsevier Science. His great passion for publishing made an immediate and lasting impression. Whether it be authoring, editing, or simply helping others to publish the best possible science, Joe was always equally enthusiastic. I was fortunate enough to work with Joe on a succession of successful publishing projects, including many major special issues for top journals, culminating in the launch of a new journal in 1998, *Environmental Science & Policy*.

This was no doubt one of the highlights of Joe's editorial work. Joe saw a gap in the available environmental literature as a result of his research interests and extensive interactions with government agencies and business. How could major environmental scientific issues be reported successfully and set against the wider socioeconomic and international perspective? To test his idea for the new journal, Joe brought to bear his very influential and extensive network of contacts worldwide. When that came out positive, and Elsevier was able to give a "thumbs up", he pulled out all the stops and had the first issue of papers ready in what seemed to be record time — supported all the way by his wife Jan, an experienced editor in her own right. Joe's conviction of the need for the new journal was soon vindicated. In the second year of publication, the new journal increased in frequency to six issues per year.

The early years building up *Environmental Science & Policy* as Editor-in-Chief were exciting and involved long hours. But all the while, Joe would find time and energy to go visiting environmental science institutes throughout Europe and the U.S., conducting his own research projects and encouraging his colleagues to publish their research, whether with his own journal or other reputable journals. Joe simply loved publishing and wanted all his colleagues to be aware of the benefit of timely and high-quality publication.

He stepped down as Editor-in-Chief of *Environmental Science & Policy* in 2001 to focus on his new business, Environmental Synergy, Inc. (ESI). When Joe explained what ESI was all about, there seemed no better business for an environmental scientist to embark on. Major corporations would pay for the

©2006 with author.
Published by TheScientificWorld, Ltd.; www.thescientificworld.com reforestation of vast tracks of low-grade agricultural land in return for carbon credits. Inevitably, a favorite topic of conversation would be how many million trees his company had planted in the last few months and how, working with conservation organizations, the planting was tailored to optimize biodiversity.

In latter years, we have had worked together on other projects, including the Second International Nitrogen Conference, where he was a driving force on the organizing committee. Joe and his colleague Carol Jordan were also chiefly responsible for the publication of the proceedings, published jointly by TheScientificWorld and Balkemar Publishers imprints (both now part of the Informa Group of companies). He was a Principal Editor for “Environmental Science and Management”, a domain of TheScientificWorldJOURNAL, that published several influential papers on climate change.

Joe had all the remarkable interpersonal and technical skills to communicate at all levels of industry and academia, and he juggled his research and business interests successfully, without forgetting his love of editing.

On a personal level, Joe was a man of integrity and faith. You always knew where you stood with Joe, whether in business or as a friend. He always had a positive outlook and never had a bad word to say about anyone. He was completely devoted to his young family, and his pride in his family was evident to all his friends who would get regular updates on the achievements and newly discovered talents of his young sons. I feel privileged to have worked with him and to have been able to call him a friend.

Joe Wisniewski, Ph.D.
1950–2006

**This article should be cited as follows:**

Vindenes Allen, A. (2006) In memory of Joe Wisniewski, Ph.D.: an outstanding editor for the environmental sciences. *TheScientificWorldJOURNAL* 6, 1144–1145. DOI 10.1100/tsw.2006.223.
